# Buckling critical pressures in collapsible tubes relevant for biomedical flows

**DOI:** 10.1038/s41598-023-36513-6

**Published:** 2023-06-08

**Authors:** Marco Laudato, Roberto Mosca, Mihai Mihaescu

**Affiliations:** Department of Engineering Mechanics, FLOW Research Center, KTH Royal Institute of Technology, 10044 Stockholm, Sweden

**Keywords:** Biomedical engineering, Fluid dynamics

## Abstract

The behaviour of collapsed or stenotic vessels in the human body can be studied by means of simplified geometries like a collapsible tube. The objective of this work is to determine the value of the buckling critical pressure of a collapsible tube by employing Landau’s theory of phase transition. The methodology is based on the implementation of an experimentally validated 3D numerical model of a collapsible tube. The buckling critical pressure is estimated for different values of geometric parameters of the system by treating the relation between the intramural pressure and the area of the central cross-section as the order parameter function of the system. The results show the dependence of the buckling critical pressures on the geometric parameters of a collapsible tube. General non-dimensional equations for the buckling critical pressures are derived. The advantage of this method is that it does not require any geometric assumption, but it is solely based on the observation that the buckling of a collapsible tube can be treated as a second-order phase transition. The investigated geometric and elastic parameters are sensible for biomedical application, with particular interest to the study of the bronchial tree under pathophysiological conditions like asthma.

## Introduction

**Figure 1:**
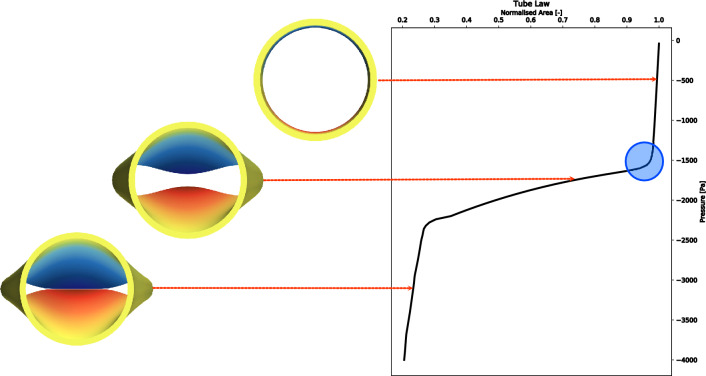
An example of tube law (on the right). The blue circle indicates the region where the buckling transitions occurs. On the left, the cross-sections relative to the pre-buckling, post-buckling, and post-contact regime are presented. The area is normalised on the of the central cross-section corresponding to the rest configuration.

The possibility to study mass transportation in the human body, either in the case of air or blood, in terms of mathematical and numerical models represents one of the most fruitful examples of the bridging between medicine and engineering. The application of Computational Fluid Dynamics (CFD), Fluid-Structure Interaction (FSI), and Aeroacoustics models has remarkably enhanced the understanding of pathophysiological conditions of the circulatory system^1^, the respiratory system^2, 3^, voice production process^4^, and the cerebrovascular system^5^, among the others. The validity of the results obtained by means of such numerical models needs to be confirmed by case-specific experimental campaigns. The variety and the geometrical complexity of the human vessels can make this crucial step extremely challenging. To this regard, simplified models like *collapsible tubes*^6–8^ are still widely employed in both numerical model and clinical practice. Despite the simplified geometry, the phenomenology of a collapsible tube is rich enough to capture the most relevant physical mechanisms of collapsed vessels^9^. The dynamics of a collapsible tube depends essentially on the so-called *intramural pressure* which is defined as the pressure difference between the interior (the *lumen*) and the exterior of the tube. In presence of fluid flow, an additional contribution due the acceleration of the flow close to the constriction, resulting in a region of negative static pressure, needs to be considered. As the external pressure increases (i.e., the intramural pressure becomes negative) the tube starts to collapse. For a critical value of the intramural pressure, the tube experiences a buckling phenomenon resulting in a two-lobes cross section (see Fig. 1). Such a value is called *buckling critical pressure* and plays a major role in the assessment and diagnosis of many pathologies involving stenosis and constrictions^10–12^. When in this configuration, small variations of the intramural pressure would result in large variations of the area of the lumen. If the external pressure keeps increasing (or internal pressure keep decreasing due to the flow accelerating), the internal walls of the tube will touch each other (see Fig. 1) and eventually leading to the complete closure of the lumen. A precise and patient-specific estimation of the buckling critical pressure allows for more informed clinical decisions. An example is given by the pharyngeal buckling critical pressure for patients affected by Obstructive Sleep Apnoea (OSA). OSA is the most common pathology in the sleep-disordered breathing spectrum^13^. Patients affected by OSA experience the recurring collapse of the pharynx during sleep, causing apnoea which severely affects the patients’ quality of life. The assessment of the severity of the pathology and the treatment choice strongly depends on the values of the buckling critical pressure of the pharynx^14^. However, its estimation requires the patients to spend the night in the hospital and being continuously monitored, resulting in a quite intrusive experience for the patient and in a high economic impact on the healthcare system^15^.

From a more quantitative perspective, this problem can be formulated in terms of the so-called *tube law*, i.e. the relation between the intramural pressure and the area of the central cross-section of the collapsible tube (see Fig. 1). It is important to remark that this relation is valid in absence of flow. The blue circle in Fig. 1 highlights the region where the transition happens. As the negative intramural pressure increases in absolute value, the transition to the buckling configuration first occurs. The hour-glass shaped cross-section area quickly reduces its value until the contact of the lumen happens. In the context of the tube law, it is possible to state the research questions as: is it possible to estimate the exact value of the buckling critical pressure in the blue area in Fig. 1 starting from the geometric and elastic properties of the collapsible tube? How these values depend on such properties? Is it possible to find general equations able to estimate the buckling critical pressure for collapsible tubes relevant for biomedical flows?

Interestingly, the problem of the buckling critical pressure was already addressed in the early 1900 by von Mises^16^ (for a treatment in English, it is possible to consult Timoshenko’s book^17^) who derived the following equation for a two-lobed buckling cross-section:

1 $$\begin{aligned} -p^{crit}_{buckl} = \frac{2E}{(1-\nu ^2)}\gamma ^3 + \frac{1}{1+ \left( \frac{4d}{\pi }\right) ^2}\left[ \frac{2E(7-\nu )}{3(1-\nu ^2)}\gamma ^3+\frac{2E}{3}\gamma \right] . \end{aligned}$$

In this equation, *E* is the Young’s modulus, $\nu$ is the Poisson ratio, $\gamma =h/D$ where *h* is the thickness of the wall and *D* is the internal diameter, and $d=l_0/D$ where $l_0$ is the length at rest of the tube. The analytical derivation of this equation, however, relies on strong geometrical assumptions. The first assumption on the perfect cylindrical geometry of the system hinders its application to more realistic geometries. Secondly, as it relies on thin shell theory, this equation overestimates the value of the critical pressure for the range of parameters of interest for biomedical flows^18^ (see “Buckling critical pressure” ). Moreover, human vessels are subject to a considerable pre-stretch^19^ (up to $60\%$ of the original length for the respiratory system^20^) that is not considered in Eq. (1). Consequently, the analysis of the tube law has been object of several studies and is currently extremely relevant from both engineering^21^ and clinical^22^ perspectives. Several analytical derivations of the tube law, under different assumptions, have been proposed. Whittaker et al.^23^ studied the dynamics of a long, pre-stretched, thin-walled collapsible tube undergoing an isotropic intramural pressure. The resulting equations are only valid under the hypothesis of small deformations. Shapiro and collaborators analysed the behaviour of the collapsible tube in presence of the steady^24^ and unsteady^25^ fluid flow, and its application to medicine^26^ and to the analysis of wave propagation phenomena^27^. Conrad proposed a lumped parameter model to describe the dynamics of a collapsible tube as a flow-controlled nonlinear resistance^28^. Another lumped parameter model was proposed by Bertram^29^ to describe the complex fluid–structure interaction behaviour of a collapsible tube. Due to the relatively simple geometry of the system, the experimental investigation of collapsible tubes has a long history. The first experimental observation of the onset of venous self-excited oscillations has been reported in 1824 by D. Barry (see the historical review by Bertram^30^ for more details). In more recent times, the non-linear dynamics of thin-walled tubes has been investigated under unsteady external pressure by Kumar and collaborators^21^. Gregory et al.^31^ have employed multiple camera stereoscopy to determine an empiric generalised tube law for thin-walled tube undergoing different axial pre-tensions. Such experimental data^32^ has been employed for the experimental validation of the results of the present work. Despite such an impressive body of work which has largely clarified many aspects of the behaviour of collapsible tubes, the estimation of the buckling critical pressure remains an open question. The effects of relevant parameters such as the axial pre-stretch, wall thickness, and tube length on the tube law have been investigated experimentally by Bertram^33^. More recently, Kozlovsky et al.^34^ employed an experimentally validated 2D numerical model to study the effects of the wall thickness on the post-buckling behaviour of a collapsible tube in absence of flow. In their work, the buckling critical pressure was estimated from the tube law by means of a graphical method which consists in finding the interception between the two linear region connected by the smooth knee at the onset of the buckling (the blue region in Fig. 1). Zarandi and collaborators have analysed the effects of the tube length on the buckling critical pressure of collapsible tubes^35^ in absence of flow. In that work, the estimation of the buckling critical pressure was performed by first obtaining a linear fit of the tube law before the buckling and then displace it by an arbitrary amount. Interestingly, their results do not qualitatively agree with Eq. (1) as they compute the dependence of the critical pressure on the length-diameter ratio as $p^{crit}_{buckl}\sim d^{-3.3}$ which further confirm the need for additional investigations.

Summarising, the two main problems that are hindering new significant advancements in this field and, consequently, the possibility to extend these analyses to clinical practice are the following:

- A careful analysis of the tube law (as the one sketched in Fig. 1) shows that the transition between the pre- and post buckling state of a collapsible tube is continuous. Consequently, the estimation of the actual critical pressure is not trivial and has been performed only by using graphic or heuristic criteria which can be hardly generalised.
- Most of the modelling approach are fundamentally dependent on strong geometric assumptions such as perfect cylindrical tubes or 1D elastic rings. Moreover, axial pre-stretched is generally neglected.

In this work, a new method based on Landau’s theory of phase transitions^36^ to determine the value of the buckling critical pressure from the analysis of the tube law is proposed. The main assumption is that the transition of a collapsible tube to the buckling state can be described as a spontaneous symmetry breaking. This hypothesis has been recently consolidated in the work by Turzi^37^ who has proven that the buckling behaviour of an elastic ring under the action of an uniform external pressure is a second order phase transition. The methodology of the present work is based on the implementation of an experimentally validated 3D numerical model of a collapsible tube. The output of these simulations consists into a set of tube laws obtained by spanning a range of values for the geometric parameters of collapsible tubes that are relevant for biomedical applications. A post-processing technique based on phase transitions theory is used to estimate the buckling critical pressure. The dependence on the geometric parameters of the buckling critical pressure obtained by using this technique is compared to Eq. (1). Finally, using the non-dimensional variables proposed by Gregory et al.^31^, a set of general equations for the buckling and contact critical pressure is presented.

This work aims at investigating the feasibility of the proposed method to estimate buckling critical pressure in the simpler case of a cylindrical collapsible tube in absence of fluid flow. This assumption is not hindering the completeness of the physical description of the problem. Indeed, one of the main advantages of this method is that it can be generalised without further assumptions to more complex cases involving FSI and realistic geometries, which will be object of a future work.

## Numerical model and methods

**Figure 2:**
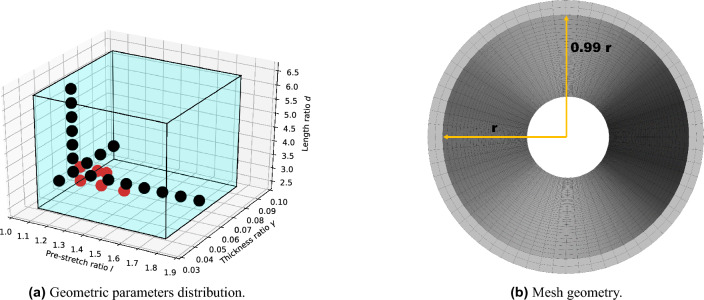
(Left panel) Distribution of the geometric parameters values investigated in this work. The black dots are relative to the simulations, while the red dots refers to the experimental data from Gregory et al.^31^. The blue box represents the space of possible values admitted in the models by Horsfield^19^ and Hoppin^20^. (Right panel) Sketch of the mesh employed in the numerical simulations. To control the direction of the buckling, the radial cross section of the tube is an ellipse with minor axis aligned with the vertical direction and long 0.99*r*.

The aim of the numerical model is to predict the displacement vector field of a collapsible tube whose external wall is subject to an inward isotropic pressure. The geometry of the tube is described in terms of three non-dimensional parameters: the length-to-diameter ratio *d*, the thickness-to-diameter ratio $\gamma$, and the ratio, namely *l*, between the length obtained after the imposition of an axial pre-stretch and the length at rest. To make the analysis relevant for human vessels, the values of these geometric parameters have been selected using the Horsfield model^19^ and the work by Hoppin^20^ (blue box in Fig. 2a). As shown in Fig. 2a, human vessels are generally characterised by a low value of *d* (i.e., they are relatively short with respect to their diameter) and a wide range of thickness values. Remarkably, the axial pre-stretch can reach values up to 60% of the original length^20^ and, therefore, cannot be neglected in the modelling scheme. The numerical simulations are performed by using the commercial software Siemens Star-CCM+. For any triplet $(d,\gamma , l)$ of geometric parameters (black dots in Fig. 2a), a 3D replica of the corresponding tube is implemented using the built-in CAD software. To control the direction of the buckling, the cross-section of the domain is designed as an ellipse with axes ratio equal to 0.99 (see Fig. 2b). Since the simulation aims at capturing the pre and post-buckling behaviour of the system, the tube is modeled as a Neo-Hookean hyperelastic material to account for such large deformations regime (see “Sensitivity analysis”  for a comparison with linear elasticity theory). The material is treated as nearly incompressible. The mesh has been generated by employing a structured volume operation, using as input surface one of the short sides of the domain. The computational domain is discretised with two radial layers and 50 longitudinal layers of hexahedral mesh cells. The angular direction is divided into 64 elements (see Fig. 2b). The choice of hexahedral elements ensures a correct estimation of large strains even with linear finite elements^38^. The time step is fixed for all the simulation and it is $\Delta t = 0.1$ s with a second-order marching scheme. Each time step is divided into 10 inner iterations of the solver to ensure the proper convergence of the solution. In the context of a purely solid mechanics simulation, the convergence is measured in terms of the residual error of the discretised version of the solved differential equations. The solver employed in this study estimates such error *r* for each element of the mesh and then computes its root mean square as $R_{rms}=\sqrt{\frac{1}{n}\sum _n r^2}$, where *n* is the number of mesh cells. This value is computed for each of the 10 inner iterations within a time step and then normalised. The number of inner iterations has been selected in such a way that the residual error at the end of each time step is of the order of $R_{rms}\sim 10^{-14}$–$10^{-16}$ which ensures an excellent convergence of the numerical solution. The boundary conditions of the problem are imposed as the following: one short side of the domain is clamped i.e., no movement is allowed to the whole cross-section. The other short side of the domain is pre-stretched by a quantity which depends on the value of the geometric parameter *l*. Successively, the position of this short side obtained as a result of the pre-stretch is kept fixed for the rest of the simulation. On the external wall of the domain (i.e., the cylindrical surface) an isotropic positive pressure is imposed, resulting in a negative intramural pressure. The value of the external pressure increases linearly in time (see “The tube law”  for more details), causing first the buckling of the tube and finally the contact of the internal wall. The contact is handled by means of a repulsive virtual plane to avoid the penetration of the wall. When the internal walls are approaching the contact, the plane exerts a force in the direction of its normal and the walls will consequently lie on the plan without penetrating each other.

In the following subsection, the post-processing method to compute the tube law is described. Successively, a sensitivity analysis in terms of grid convergence study, boundary conditions, and modelling choices is presented. Finally, the experimental validation of the numerical results is discussed.

### The tube law

The tube law describes the relation between the intramural pressure and the area of the central cross-section of a collapsible tube. The intramural pressure is defined as the difference between the internal and external pressure, namely:

2 $$\begin{aligned} p_{intr} = p_{int} - p_{ext}. \end{aligned}$$

Due to the absence of fluid flow, the intramural pressure is only determined by the value of the external pressure, as the gauge pressure is set to zero and $p_{int}=0$. The external pressure is isotropic and increases linearly in time according to the following relation

3 $$\begin{aligned} p_{ext}(t) = \frac{p_{max}}{\tau } t, \end{aligned}$$

where $p_{max}>0$ is the maximum value of the external pressure and $t\in [0,\tau ]$. A sensitivity study in terms of the ratio $p_{max}/\tau$ is discussed in “Sensitivity analysis” . At each time step, the value of $p_{intr} = -p_{ext}$ is recorded. The central cross-section of the tube will be the most collapsed since it is the furthest radial cross-section from the constrained faces. To determine the value of the area, the radial coordinates $(r_i^j, \vartheta _i^j)$ of the corresponding deformed perimeter are recorded at each time-step, where the index $i=1,\dots ,N$ labels the mesh elements and *N* is the total number of mesh elements on the perimeter. The index $j=1,\dots ,M$ corresponds to the *j*-th time step and *M* is the total number of time steps needed for the external pressure to reach the value $p_{max}$ in Eq. (3). The area $A^j$ of the central cross-section can be then computed as:

4 $$\begin{aligned} A^j = \frac{1}{2} \sum _{i=1}^{N-1} \left( r_i^j\right) ^2\left| \vartheta _{i+1}^j-\vartheta _i^j\right| . \end{aligned}$$

The two sets $\{p_{intr}^j\}_{j=1}^M$, $\{A^j\}_{j=1}^M$ define a tube law. For each triplet of geometric parameter $(d,\gamma ,l)$ indicated in Fig. 2, a tube law is computed using Eq. (4) from the corresponding numerical simulation. In “Buckling critical pressure” , a post-processing technique based on phase-transition theory will be introduced to determine the value of the buckling critical pressure from the tube law.

### Sensitivity analysis

**Figure 3:**
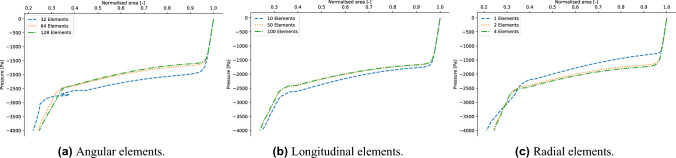
Mesh sensitivity analysis of the numerical model.

A grid convergence analysis is required to determine the most efficient mesh able to obtain reliable results. The study is performed by changing the number of mesh elements in the radial, angular, and longitudinal directions. Details on the mesh specifications can be found in Table 1. The evaluation of the different grids is performed by comparison of the corresponding tube laws. The results are shown in Fig. 3. As it is particularly evident from the analysis of the angular elements (see Fig. 3a), the coarse mesh is not correctly predicting the buckling transition and becomes unstable in the contact region. For all the following analysis, the intermediate mesh has been employed. With this choice, the simulation time on 128 cores is approximately fifteen minutes.

**Table 1:** Specifics of the mesh sensitivity analysis.

Angular mesh elements			Longitudinal mesh elements			Radial mesh elements		
Ang = 16	Long = 50	Rad = 2	Ang = 32	Long = 10	Rad = 2	Ang = 32	Long = 50	Rad = 1
Ang = 32	Long = 50	Rad = 2	Ang = 32	Long = 50	Rad = 2	Ang = 32	Long = 50	Rad = 2
Ang = 128	Long = 50	Rad = 2	Ang = 32	Long = 100	Rad = 2	Ang = 32	Long = 50	Rad = 4

The angular, longitudinal, and radial mesh analyses have been performed separately. The numbers in the tables represent the number of elements in each direction.

**Figure 4:**
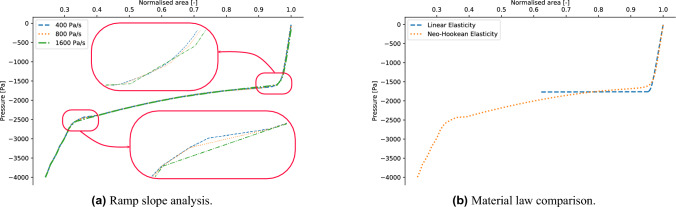
(Left panel) Analysis of the dependence of the model on the external pressure ramp. (Right panel) Comparison of the tube laws obtained by employing linear elasticity and Neo-Hookean elasticity model.

To ensure that the computation of the tube law is not influenced by the slope of the ramp for the external pressure described by Eq. (3), a sensitivity analysis in terms of the ratio $p_{max}/\tau$ is performed. In particular, by fixing the value of $p_{max}=4000$ Pa, three different values of $\tau \in (10 \, \text { s}, 5 \, \text { s}, 2.5 \, \text { s})$ are investigated, corresponding to pressure rates of $(400 \, \text { Pa/s}, 800 \, \text { Pa/s}, 1600 \, \text { Pa/s})$, respectively. The results are interpreted in terms of the corresponding tube laws. The value of $p_{max}$ has been chosen to be relevant for the analysis of pathological respiratory conditions. Indeed, in normal breathing conditions the pressure drop between the alveolar ducts and the mouth is of the order of 2000 Pa^39^. However, in case of forcing expiration it can vary from 4000 to 10000 Pa^40^, depending on the size of the patient^41^. In the case of asthma, patients often show forced expiration with presence of wheezing in this range of pressures, which is connected to the collapse of the lungs airways^42^. As shown in Fig. 4a, the pressure resolution associated with a rate of pressure of 1600 Pa/s is too coarse to capture the behaviour of the tube in both the buckling and contact phase. For all the following analysis, the largest value of $\tau$, corresponding to 400 Pa/s is used.

**Figure 5:**
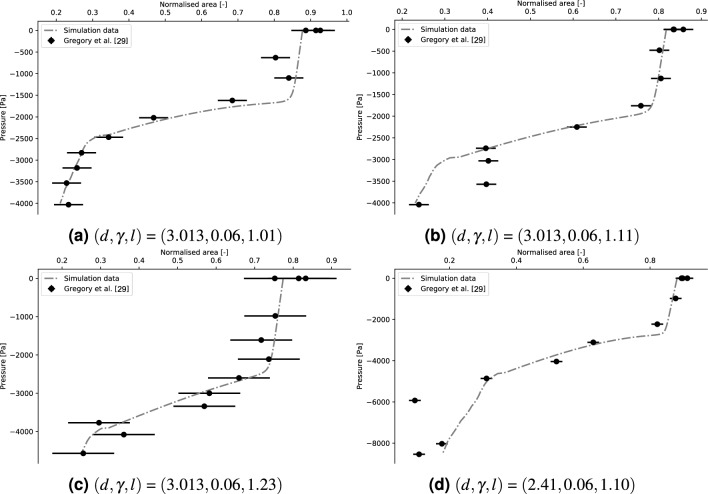
Validation of the numerical model by comparison with experimental data^31, 32^. To estimate the error bars, the initial area is measured 3 times. The length of the error bars is equal to the absolute difference between the corresponding maximum and minimum values.

To account for the large deformations involved in the full pre- and post-buckling behaviour of a collapsible tube, a hyperelastic material needs to be employed^43^. In this analysis, a Neo-Hookean material law has been implemented. The corresponding strain energy potential reads

5 $$\begin{aligned} \Psi = \chi \left( I_1-3\right) +\frac{\lambda }{2}(J-1)^2, \end{aligned}$$

where $I_1$ is the trace (first invariant) of the right Cauchy–Green deformation tensor, *J* is the determinant of the deformation gradient tensor, and

6 $$\begin{aligned} \chi = \frac{E}{4(1+\nu )},\quad \lambda =\frac{\nu E}{(1-2\nu )(1+\nu )}, \end{aligned}$$

where $\nu$ and *E* are the Poisson’s ratio and the Young’s modulus, respectively. The value of these two elastic parameters are $\nu =0.49$ and $E=1$ MPa. The density of the tube is $\rho =1000$ kg m$^{-3}$. These values have been estimated in the work by Gregory^31^ to be relevant for the analysis of conduits in human lungs. As discussed in the next subsection, the Neo-Hookean model is able to reproduce the experimental results. The comparison between the tube laws obtained with a Neo-Hookean material and an isotropic linear elastic material is presented in Fig. 4b. By using the linear elastic model the simulation becomes unstable and unable to capture the tube law in the post-buckling region. The reason is to be found in the large deformations involved in the process. Although it might be of interest to study the sensitivity of the obtained results in terms of other hyperelastic laws^44–46^, the employment of Neo-Hookean elasticity has shown a remarkable numerical convergence (see “Numerical model and methods” ) and it is able to reproduce the experimental results (see Fig. 5). Moreover, other material laws might require additional parametrisation analysis with respect to Neo-Hookean law, whose coefficients can be directly computed from the Poisson ratio and the Young’s modulus using Eq. (6). Consequently, Neo-Hookean elasticity will be employed in the rest of the treatment and the comparison of the results with respect to different material laws will be the object of a future work.

### Validation

The experimental validation of the results obtained by the numerical model is performed by comparison with the (publicly available) experimental data provided by Gregory^31, 32^. In the experimental rig employed, a collapsible tube is first pre-stretched and then clamped. The value of the intramural pressure is lowered using a syringe and monitored with a manometer. The corresponding values of the area of the central cross section are computed by analysing the images taken by a camera system from multiple positions (more details can be found in the original paper^31^).

The validation is performed by comparing the tube laws obtained experimentally and by the numerical model. For each tube employed in the comparison, a digital replica (represented by the red dots in Fig. 2a) is implemented in Star-CCM+ using the built-in CAD software. The same boundary conditions (axial pre-stretch and clamping) are imposed. The intramural pressure range explored with the current simulations is larger and includes the corresponding one employed in the experiments. In Fig. 5, the comparison with four different tube geometries and pre-stretch values are reported. The pre- and post-buckling behaviour of the system is well captured by the numerical model.

## Buckling critical pressure

In this section, a method to determine the value of the buckling critical pressure from the tube law is presented. The main assumption is that the buckling phenomenon of a collapsible tube is a spontaneous symmetry breaking and, therefore, can be treated as a second-order phase transition. A second-order phase transition occurs when the system *continuously* (and not instantaneously) reaches a new state of reduced symmetry^47^. The phenomenological observation that an isotropic external pressure generates a buckled cross-section shape which is not rotational invariant endorses this hypothesis. Moreover, as evident from the tube law (see the blue circle in Fig.1), the transition from the pre- to the post-buckling state occurs continuously. In a more rigorous fashion (and inspired by biophysical problems), Turzi^37^ has demonstrated that the buckling phenomenon of an elastic stretchable ring undergoing an isotropic pressure is a second order phase transition. Although the rigorous transliteration of these results obtained on a 1D ring to a 3D pre-stretched collapsible tube is beyond the scope of this work, the phenomenological analogy between the buckling behaviour of a ring and of the central cross-section of a collapsible tube has been widely established^48^. This analogy has been exploited in several occasions to extend 1D analytical results to the treatment of 3D collapsible tubes^49, 50^. In the economy of the present work, however, this observation serves only to the scope of further endorsing the method’s main assumption i.e., that the collapsible tube’s buckling is a second order phase transition. The methodology described in the next subsection, indeed, does not require any geometrical simplification and can be extended without further hypothesis to realistic geometries inspired by the human conduits and vessels associated with the respiratory and circulatory systems, even in presence of fluid flow.

### Estimation of the buckling critical pressure

The thermodynamic treatment of second-order phase transitions requires the introduction of two main mathematical objects: the order parameter, $\alpha$, and the Landau potential, $\varphi$, of the system^36^ (a common example of Landau potential is the free energy of the system). The order parameter is a function of some thermodynamic variable which is different from zero before the transition and equal to zero after the transition. One typical example is the magnetization as a function of the temperature in the ferromagnetic–paramagnetic transition. The main assumption of this mean-field theory formulation is the absence of long-range correlations in the domain. Moreover, the Landau potential is required to respect the same symmetries of the differential equations which describe the evolution of the system and to be analytic in the order parameter and its gradient^36^. Consequently, in the transition region, the Landau potential can be Taylor expanded in terms of the order parameter $\alpha$ as

7 $$\begin{aligned} \varphi (\alpha ) - \varphi _0 = c_1(\xi -\xi _{crit})\alpha ^2 + \frac{c_2}{2}\alpha ^4, \end{aligned}$$

where $c_1>0$ and $c_2 >0$ are constants, $\xi$ is a thermodynamic variable, and $\xi _{crit}$ is the critical value for $\xi$ at which the transition occurs. The minimization of the Landau potential yields an equation for the order parameter:

8 $$\begin{aligned} \frac{\partial \varphi }{\partial \alpha } = 2c_1(\xi -\xi _{crit})\alpha +2c_2\alpha ^3=0. \end{aligned}$$

**Figure 6:**
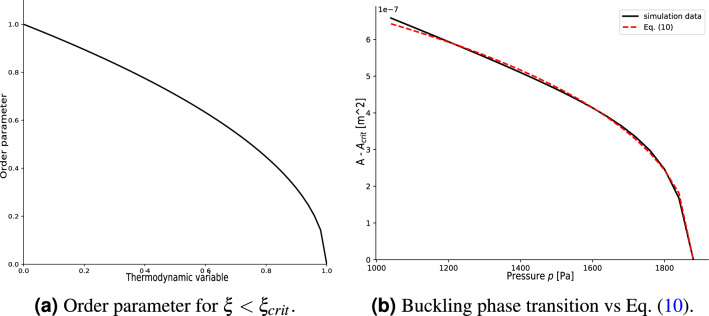
(Left panel) Plot of Eq. (9) for $\xi _{crit}=1$ and $\xi <\xi _{crit}$. (Right panel) Detail of the pre-processed buckling transition region in the tube law fit with Eq. (10). The analogy between the two plots endorses the validity of the hypothesis.

This equation has two solutions (see Fig. 6a):

9 $$\begin{aligned} \alpha = {\left\{ \begin{array}{ll} \left[ -\frac{c_1}{c_2}(\xi -\xi _{crit})\right] ^{1/2},&{}\xi <\xi _{crit}\\ 0,&{}\xi \ge \xi _{crit} \end{array}\right. }. \end{aligned}$$

Therefore, if the relation between the order parameter $\alpha$ and the thermodynamic variable $\xi$ in a small neighborhood of the transition is known, it is possible to estimate the value of $\xi _{crit}$ by means of Eq. (9). The main limitation of this approach is that it provides a purely phenomenological description of the transition, neglecting for instance the treatment of fluctuations. Nonetheless, it can be effectively used to investigate the phase transition’s critical points, which is exactly the scope of this work. An additional layer of complexity needs to be accounted for when considering elasticity problems. Although the a priori known deformation energy of the system can play the role of a Landau potential, this is not a function anymore but a functional defined on an infinite dimensional space. In the work by Turzi^37^, he performed a rigorous reduction of the elastic energy to a finite dimensional Landau potential to study buckling phenomena of many elastic systems, including a 1D extensible ring undergoing an external isotropic pressure. Moreover, he shows that the enclosed area of the ring is a function of the order parameter. These observations can be used in the context of this work to estimate the buckling critical pressure of a collapsible tube from the tube law. Following Turzi, the area of the central cross section of the tube can be used as a representative function of the order parameter. The relation between the order parameter and the intramural pressure $p_{intr}$ (which plays the role of the variable $\xi$ in Eq. (9)) can be therefore analysed using the tube law. The methodology consists into a fitting procedure of the tube law with a generalisation of Eq. (9) able to fit a wider portion of the tube law, as this relation is valid only in a small neighborhood of the transition. Among the several extension of Eq. (9) proposed in literature^51^, the following function has shown the best results (see Fig. 6b)

10 $$\begin{aligned} A - A_{crit} = c_1\tanh \left( c_2\left( \frac{\tilde{p}_{crit} - \tilde{p}}{\tilde{p}}\right) ^\beta \right) \end{aligned}$$

where $A_{crit}$ is the value of the area corresponding to the buckling critical pressure $p_{crit}=-\tilde{p}_{crit}$, $c_1>0$ and $c_2>0$ are two free parameters, $\tilde{p}=-p_{intr}$, and $\beta >0$ is an additional free parameter, usually called critical exponent. The results of this analysis have been obtained according to the following procedure:

1. For any triplet $(d,\gamma , l)$ of geometric parameters (i.e., for any data point in Fig. 2a), the corresponding numerical model is implemented according to “ Numerical model and methods”.
2. The corresponding tube law, i.e. the two sets $\{p_{intr}^j\}_{j=1}^M$, $\{A^j\}_{j=1}^M$, is computed as discussed in “The tube law” .
3. The tube law is pre-processed. Since the present analysis focuses on the buckling pressure, the region of the tube law concerning the contact is neglected. Moreover, the intramural pressure is redefined in terms of $\tilde{p}=-p_{intr}$. An example of the corresponding plot is shown as the solid black line in Fig. 6b.
4. A Python algorithm employing the scipy.optimize.curve_fit function^52^ is used to fit the pre-processed tube law with Eq. (10) (dashed red line in Fig. 6b). The value of $A_{crit}$ is optimised to maximise the quality of the fit. The values of the parameters $(c_1, c_2, \tilde{p}_{crit}, \beta )$ with the corresponding variances are extracted.

In the following subsection, the results of this analysis and the dependence of the buckling critical pressure on the geometric parameters are discussed.

### Dependence on the geometric parameters

**Figure 7:**
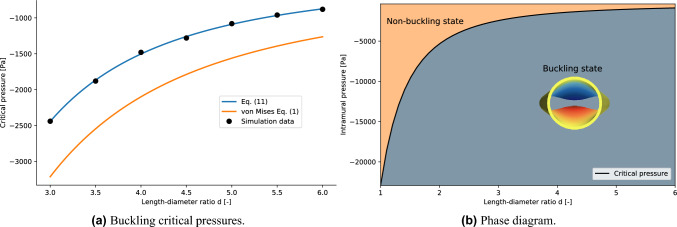
(Left panel) Comparison between the simulation data fit with Eq. (11) and the von Mises equation. (Right panel) Phase diagram for the buckling phase transition in terms of the length-diameter ratio *d*.

**Figure 8:**
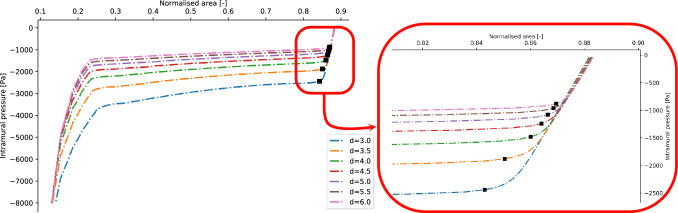
Tube laws obtained by spanning the value of the length-diameter ratio *d*. The black dots represent the value of the buckling critical pressures and the corresponding areas estimated for the different tube laws.

The algorithmic procedure introduced in the previous section allows for the study of the dependence of the buckling critical pressure on the geometric parameters of a collapsible tube. In order to capture the full post-contact behaviour of the system, the maximum intramural pressure is fixed at $p_{max}=8000$ Pa and $\tau =20$ s, corresponding to a resolution in pressure of 40 Pa (see Eq. (3)). Firstly, the dependence on the length-to-diameter ratio *d* is investigated. The numerical values of the parameters $(c_1, c_2, \tilde{p}_{crit}, \beta )$ obtained by the fitting procedure and the corresponding variance can be found in the supplementary data. The average value of the critical exponents is $\bar{\beta }=0.55\pm 0.07$, which is consistent with the expected value of the exponent of Eq. (9). The values of the critical pressures obtained for $d\in (3,3.5,4,4.5,5,5.5,6)$ are shown in Fig. 7a. The relation between $p_{crit}$ and the length-to-diameter ratio *d* is obtained by a fitting procedure using the following function

11 $$\begin{aligned} -p_{crit}(d) = Ad^{B} + C \end{aligned}$$

where A, B, C are free parameters. This model function mimics the same functional dependence of $p_{crit}$ on *d* described by Eq. (1) and in the work by Zarandi^35^. The resulting value are listed in Table 2. The other geometric parameters are fixed to $l=1.1$ and $\gamma =0.06$. Interestingly, the exponent *B* obtained with the presented method almost matches within the error with the one in the von Mises equation (1), whereas Zarandi estimated a value of  -3.3. However, as shown from Fig. 7a, Eq. (1) overestimates the value of the buckling critical pressure in the range of parameters of interest for human vessels. The differences with respect to the results obtained by Zarandi can be probably traced back to the heuristic definition of the buckling critical pressure employed in his work^35^. In the context of phase transition theory, Eq. (11) evaluated using the parameters listed in Table 2 defines the boundary between the non-buckling state and the buckling state for the collapsible tube in terms of the length-diameter ratio (see Fig. 7b for the corresponding phase diagram $(d, p_{intr})$). A visualisation of the tube laws analysed by spanning the parameter *d* is available in Fig. 8.

**Figure 9:**
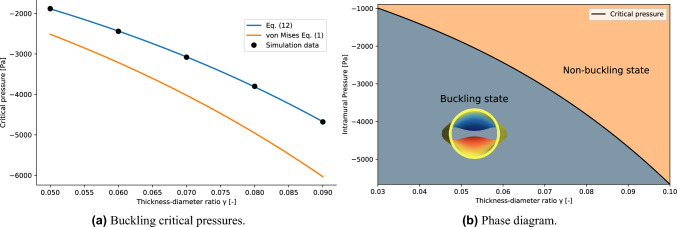
(Left panel) Comparison between the simulation data fit with Eq. (12) and the von Mises equation. (Right panel) Phase diagram for the buckling phase transition in terms of the thickness-diameter ratio $\gamma$.

**Figure 10:**
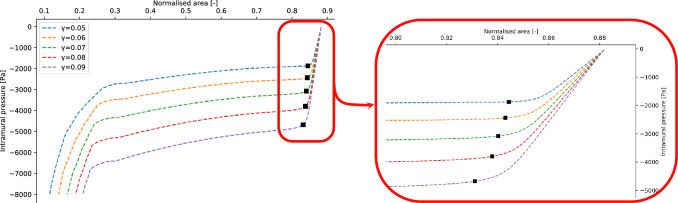
Tube laws obtained by spanning the value of the thickness-diameter ratio $\gamma$. The black dots represent the value of the buckling critical pressures and the corresponding areas estimated for the different tube laws.

Let now consider the dependence of the buckling critical pressure on the thickness-to-diameter ratio $\gamma$. The numerical values of the parameters $(c_1, c_2, {\tilde{p}}_{crit}, \beta )$ of the fit with Eq. (10) and the corresponding variances are listed in the supplementary data. The average value of the critical exponents is $\bar{\beta }=0.5\pm 0.04$, which is consistent with Eq. (9). The values of the critical pressures obtained for $\gamma \in (0.05, 0.06,0.07,0.08,0.09)$ are shown in Fig. 9a. The relation $p_{crit}=p_{crit}(\gamma )$ is obtained by a fitting procedure using the following function

12 $$\begin{aligned} -p_{crit}(\gamma ) = A\gamma ^3+B\gamma \end{aligned}$$

where A and B are free parameters. This model function follows the same functional dependency for $\gamma$ described in Eq. (1). The resulting value are listed in Table 2. The other geometric parameters are fixed to $l=1.1$ and $d=3$. The comparison between the von Mises model and the results obtained in this work is shown in Fig. 9a. For the geometric parameters values investigated in this work (see Fig. 2a), von Mises equation clearly overestimates the value of the buckling critical pressure. The reason is to be found in the assumption of the von Mises model which employs thin shell theory. Indeed, the discrepancy increases for higher values of $\gamma$. Similarly as in the previous analysis, Eq. (12) defines the boundary between the buckling state and the non buckling state in the $(\gamma , p_{intr})$ phase diagram (see Fig. 9b). A visualisation of the tube laws analysed by spanning the parameter $\gamma$ is available in Fig. 10.

**Figure 11:**
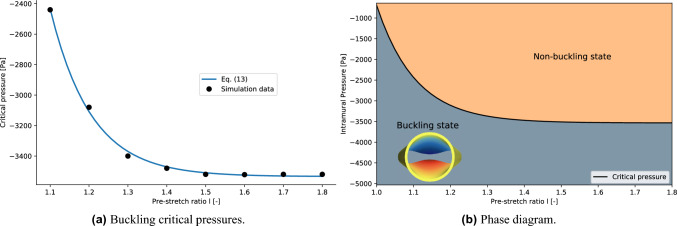
(Left panel) Comparison between the simulation data and Eq. (13). (Right panel) Phase diagram for the buckling phase transition in terms of the pre-stretch ratio *l*.

**Figure 12:**
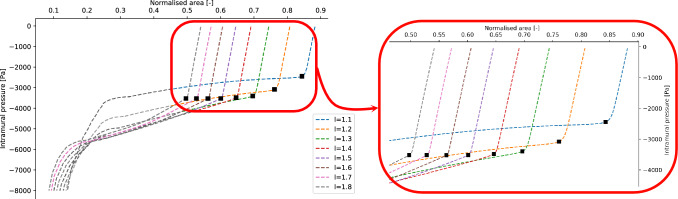
Tube laws obtained by spanning the value of the pre-stretch ratio *l*. The black dots represent the value of the buckling critical pressures and the corresponding areas estimated for the different tube laws.

Finally, the dependence of the buckling critical pressure on the axial pre-stretch length to diameter ratio *l* is analysed. The numerical values of the parameters $(c_1, c_2, \tilde{p}_{crit}, \beta )$ of the fit with Eq. (10) and the corresponding variances are listed in the supplementary data. The average value of the critical exponents is $\bar{\beta }=0.55\pm 0.05$, which is again consistent with the exponent of Eq. (9). The values of the critical pressures obtained for $l\in (1.1,1.2,1.3,1.4,1.5,1.6,1.7,1.8)$ are shown in Fig. 11a. The relation $p_{crit}=p_{crit}(l)$ is obtained by a fitting procedure using the following function

13 $$\begin{aligned} -p_{crit}(l) = A\tanh \left( Bl\right) + C \end{aligned}$$

where A, B, C are free parameters. This function has been chosen by observing that the value of the critical pressure becomes asymptotically constant for higher value of *l*. The resulting values are listed in Table 2. The dependence of the buckling critical pressure on the non-dimensional axial pre-stretch *l* is presented in Fig. 11a. The $(l, p_{intr})$ phase diagram is shown in Fig. 11b, where the boundary between the buckling state and the non-buckling state is defined by Eq. (13). A visualisation of the tube laws analysed by spanning the parameter *l* is available in Fig. 12. The effect of the pre-stretch is evident at low intramural pressures, as the initial value of the normalised area becomes smaller for larger values of the pre-stretch parameter *l*. The interesting behaviour which happens in the contact region (around $-6000$ Pa) will be object of future investigations.

**Table 2:** Values of the parameters obtained from the fit of the buckling critical pressures with Eqs. (11–13).

Parameters Eq. (11): *d*-dependence	Parameters Eq. (12): $\gamma$-dependence	Parameters Eq. (13): *l*-dependence
A = $(2.25\pm 0.29)\times 10^4$ Pa	A = $(2.53\pm 0.04)\times 10^6$ Pa	A = $(1.90\pm 1.04)\times 10^7$ Pa
B = $-2.20\pm 0.14$	B = $(3.14\pm 0.02)\times 10^4$ Pa	B = $4.75\pm 0.25$
C = $(4.37\pm 0.65)\times 10^2$ Pa	-	C = $(-1.90\pm 1.03)\times 10^7$ Pa

## General non-dimensional equations

**Figure 13:**
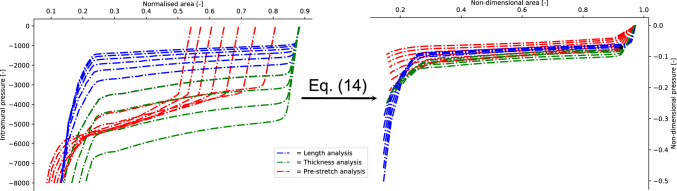
(Left panel) The set of tube laws analysed in this work. (Right panel) The corresponding non-dimensional tube laws obtained by the transformation in Eq. (14). The plots almost collapse in a single line. The black dot indicates the average values of the non-dimensional buckling critical pressure and the corresponding area as in Eq. (16).

The results described in “Buckling critical pressure”  outline the functional dependencies between the physical quantities used to describe the pre and post-buckling behaviour of a collapsible tube. The absolute values of the buckling critical pressure presented, however, depend on the geometric parameters that are kept fixed during the different analyses. In this section, a set of general equations able to estimate the values of the buckling critical pressure, together with the corresponding area, for a collapsible tube of (reasonably) arbitrary geometry are derived. The method is based on an observation by Gregory et al.^31^ who experimentally derived a set of non-dimensional variables able to approximately collapse different tube laws onto a single curve. They proved that by redefining the intramural pressure, $p_{intr}$, and the area of the central cross section, *A*, in the following non-dimensional form

14 $$\begin{aligned} \hat{p}_{intr}=\frac{p_{intr}}{E/(1-\nu ^2)}l^{-2}\gamma ^{-1}d,\quad \hat{A}=\frac{A}{\pi r^2}l \end{aligned}$$

different tube laws tend to collapse into a single non-dimensional general tube law (see Fig. 13). The validity of this claim has been experimentally proven for the range of parameters $(d,\gamma ,l)$ of interest for biomedical flows^31^.

By employing the non-dimensional variables defined in the expression (14), the following procedure has been implemented to determine a set of general non-dimensional equations for the buckling critical pressure and the corresponding area:

- For any triplet $(d,\gamma ,l)$ of geometric parameters, the corresponding numerical model is implemented according to “Numerical model and methods”.
- The corresponding tube law, i.e. the two sets $\{p_{intr}^j\}_{j=1}^M$, $\{A^j\}_{j=1}^M$, is computed as discussed in “The tube law”.
- The value of the buckling critical pressure and the corresponding area is estimated from the tube law using the procedure outlined in “Estimation of the buckling critical pressure” .
- The values of the buckling critical pressures and the corresponding areas, are redefined according to Eq. (14).

In this way, the non-dimensional values of the buckling critical pressures and the corresponding areas are computed for the whole set of geometric parameters investigated in this study. In other words, the output of this procedure is the following two sets of values

15 $$\begin{aligned} \left\{ \hat{p}_{buckl}^i\right\} _{i=1}^N,\quad \left\{ \hat{A}_{buckl}^i\right\} _{i=1}^N,\quad \end{aligned}$$

corresponding to the set of non-dimensional buckling critical pressure and buckling critical areas, respectively. The index *i* runs on the triplets of geometric parameters analysed in this study and $N=20$ indicates their total number. As shown in Fig. 13, the tube laws obtained by means of Eq. (14) do not collapse exactly into one line, instead they show a relatively small distribution of values. By computing the means and the corresponding standard deviations for each set in Eq. (15) it is possible to obtain the following expressions for the buckling critical pressure and critical area:

16 $$\begin{aligned} \frac{p^{crit}_{buckl}}{E/(1-\nu ^2)}l^{-2}\gamma ^{-1}d=-0.07\pm 0.011;\qquad \frac{A^{crit}_{buckl}}{\pi r^2}l= 0.924\pm 0.016 \end{aligned}$$

Given any triplet of geometric parameters $(d,\gamma , l)$, Eq. (16) estimate the value of the buckling critical pressures and the corresponding area.

## Conclusions

In this work, a systematic study of the buckling critical pressure of a collapsible tube has been performed by means of validated 3D numerical simulations and a post-processing technique based on phase transition theory. The functional dependence of such critical pressures in terms of the geometric parameters has been presented. Finally, a set of general non-dimensional equations for the estimation of the buckling critical pressures, together with the corresponding areas of the central cross-section of the tube, has been derived. The methodology presented in this work allows for a rigorous and reproducible estimation of the buckling critical pressure of a collapsible tube. The main advantage is that it does not require any geometric assumption but it is solely based on the observation that the buckling of a collapsible tube can be treated as a second order phase transition, making the method suitable for other kinds of applications^53, 54^. This implies that this approach can be directly employed to estimate the buckling critical pressure in complex patient-specific geometries such as the pharyngeal airways of patients with sleep apnoea, even in presence of fluid flow, which represents the natural continuation of this work. Another interesting perspective is the possibility to analyse the sensitivity of the presented results in terms of other hyperelastic theories. Finally, an in depth analysis of the contact critical pressure will objective for a future work.

## Supplementary Information

Supplementary Information.

## Data Availability

The algorithms employed in this work can be found at https://github.com/MarcoLaud/CollapsibleTube. The simulation data can be provided under reasonable request by contacting the corresponding author of this work.
