# Supplementary material for: Buckling critical pressures in collapsible tubes relevant for biomedical flows
Source: Sci Rep. 2023 Jun 8;13:9298. doi: 10.1038/s41598-023-36513-6 (PMC10250313; doi:10.1038/s41598-023-36513-6)
Supplement: Supplementary file 1 — Supplementary Information. [file 41598_2023_36513_MOESM1_ESM.pdf]

Supplementary material to the main manuscript

Marco Laudato[1,*], Roberto Mosca[1], and Mihai Mihaescu[1]

[1]KTH Royal Institute of Technology, FLOW Research Center, Department of Engineering Mechanics, Stockholm, SE-10044, Sweden.
[*]laudato@kth.se

ABSTRACT

The goal of this material is to list the values of the parameters $(c_1, c_2, \tilde{p}_{crit}, \beta)$ in Eq. (10) obtained by fitting the tube law for different values of the geometric parameters (d, γ, l). Moreover, the fitting procedure is discussed in more details.

1 Fitting the order parameter function

Given a set of geometric parameters (d, γ, l), the corresponding tube law is calculated from the numerical simulation as described in Sec. 1.1. The tube law is defined as the two sets $\{p^j_{intr}\}^M_{j=1}$, $\{A^j\}^M_{j=1}$, where the index $j = 1, \ldots, M$ represents the j-th time-step. The intramural pressure is redefined as $\tilde{p} = -p_{intr}$ to comply with the function in Eq. (10). It would be possible to redefine Eq. (10) instead but the physical meaning of description would be the same. The left hand side of Eq. (10) defines the order parameter as $A - A_{crit}$. This choice is justified by the definition of the order parameter which requires this function to vanish at the transition. The consequence is that that, during the fitting procedure, the value of A_{crit} is not a free parameter but is instead treated as a constant value. In order to determine the value of A_{crit}, an optimization procedure is employed where the optimal value of A_{crit} is defined as the one which corresponds to the minimum values of the standard deviation of the parameters $(c_1, c_2, \tilde{p}_{crit}, \beta)$. More schematically, the following procedure is employed:

- The value of A_{crit} is fixed.
- The points from the tube law in $\{p^j_{intr}\}^M_{j=1}$, $\{A^j\}^M_{j=1}$ corresponding to $A < A_{crit}$ are neglected.
- The intramural pressure is redefined as $\tilde{p} = -p_{intr}$.
- The fit of the resulting curve with Eq. (10) yields the parameters $(c_1, c_2, \tilde{p}_{crit}, \beta)$ together with the corresponding variances.
- The standard deviations of the fit parameters is computed by taking the square root of the diagonal elements of the covariance matrix.

The same procedure is employed for different values of A_{crit} until the minimum value of the standard deviations of the fit parameters is obtained. In the following, the resulting values of the fit parameters are listed for the different analysis discussed in this work.

Let consider the length-diameter ratio analysis. Its values span in $d \in (3, 3.5, 4, 4.5, 5, 5.5, 6)$ while $\gamma = 0.06$ and $l = 1.1$. The values of the parameters $(c_1, c_2, \tilde{p}_{crit}, \beta)$ in Eq. (10) are listed in Tab. 1.

d [-]	A_{crit} [10^{-5} m]	c_1 [10^{-7} m]	c_2 [-]	$\tilde{p}_{crit}$ [10^3 Pa]	β [-]
3.0	2.38	8.05 ± 0.3	1.20 ± 0.08	2.44 ± 0.04	0.48 ± 0.02
3.5	2.40	6.68 ± 0.4	1.24 ± 0.13	1.88 ± 0.04	0.46 ± 0.02
4.0	2.43	4.80 ± 0.1	1.13 ± 0.05	1.48 ± 0.04	0.56 ± 0.01
4.5	2.43	4.97 ± 0.2	1.21 ± 0.1	1.24 ± 0.04	0.47 ± 0.02
5.0	2.44	3.29 ± 0.1	1.31 ± 0.1	1.08 ± 0.04	0.59 ± 0.02
5.5	2.45	2.97 ± 0.2	1.23 ± 0.1	0.96 ± 0.04	0.57 ± 0.03
6.0	2.46	3.13 ± 0.07	1.08 ± 0.05	0.88 ± 0.04	0.56 ± 0.02

Table 1. Values of the fit parameters $(c_1, c_2, \tilde{p}_{crit}, \beta)$ for the analysis of the length-diameter ratio d.

Let consider the thickness-diameter ratio analysis. Its values span in $\gamma \in (0.05, 0.06, 0.07, 0.08, 0.09)$ while $d = 3$ and $l = 1.1$. The values of the parameters $(c_1, c_2, \tilde{p}_{crit}, \beta)$ in Eq. (10) are listed in Tab. 2.

γ [-]	A_{crit} [10^{-5} m]	c_1 [10^{-7} m]	c_2 [-]	$\tilde{p}_{crit}$ [10^3 Pa]	β [-]
0.05	2.39	7.89 ± 0.3	1.25 ± 0.1	1.88 ± 0.04	0.44 ± 0.02
0.06	2.38	8.05 ± 0.3	1.20 ± 0.08	2.44 ± 0.04	0.48 ± 0.02
0.07	2.38	8.62 ± 0.2	1.16 ± 0.06	3.08 ± 0.04	0.51 ± 0.01
0.08	2.37	9.19 ± 0.3	1.08 ± 0.05	3.80 ± 0.04	0.53 ± 0.01
0.09	2.34	10.7 ± 0.2	1.11 ± 0.05	4.68 ± 0.04	0.51 ± 0.01

Table 2. Values of the fit parameters $(c_1, c_2, \tilde{p}_{crit}, \beta)$ for the analysis of the thickness-diameter ratio γ.

Let consider the pre-stretch ratio analysis. Its values span in $l \in (1.1, 1.2, 1.3, 1.4, 1.5, 1.6, 1.7, 1.8)$ while $d = 3$ and $\gamma = 0.06$. The values of the parameters $(c_1, c_2, \tilde{p}_{crit}, \beta)$ in Eq. (10) are listed in Tab. 3.

l [-]	A_{crit} [10^{-5} m]	c_1 [10^{-7} m]	c_2 [-]	$\tilde{p}_{crit}$ [10^3 Pa]	β [-]
1.1	2.38	8.05 ± 0.3	1.20 ± 0.08	2.44 ± 0.04	0.48 ± 0.02
1.2	2.15	9.16 ± 0.3	1.17 ± 0.08	3.08 ± 0.04	0.51 ± 0.02
1.3	1.96	9.92 ± 0.5	1.01 ± 0.07	3.40 ± 0.04	0.51 ± 0.01
1.4	1.83	10.7 ± 1.2	0.43 ± 0.004	3.48 ± 0.04	0.55 ± 0.01
1.5	1.70	11.4 ± 0.1	0.68 ± 0.01	3.52 ± 0.04	0.56 ± 0.01
1.6	1.59	10.7 ± 0.1	0.70 ± 0.006	3.52 ± 0.04	0.60 ± 0.01
1.7	1.49	11.8 ± 0.1	0.62 ± 0.03	3.52 ± 0.04	0.57 ± 0.01
1.8	1.41	15.9 ± 0.2	0.49 ± 0.05	3.52 ± 0.02	0.52 ± 0.01

Table 3. Values of the fit parameters $(c_1, c_2, \tilde{p}_{crit}, \beta)$ for the analysis of the pre-stretch ratio l.
